# H3K27 demethylases are dispensable for activation of Polycomb-regulated injury response genes in peripheral nerve

**DOI:** 10.1016/j.jbc.2021.100852

**Published:** 2021-06-04

**Authors:** Phu Duong, Ki H. Ma, Raghu Ramesh, John J. Moran, Seongsik Won, John Svaren

**Affiliations:** 1 Waisman Center, University of Wisconsin-Madison, Madison, Wisconsin, USA; 2 Cellular and Molecular Pathology Graduate Program, University of Wisconsin-Madison, Madison, Wisconsin, USA; 3 Department of Comparative Biosciences, University of Wisconsin-Madison, Madison, Wisconsin, USA

**Keywords:** Polycomb, demethylase, histone, Schwann, nerve, injury, PRC, Polycomb repressive complex, SUZ12, suppressor of zeste 12

## Abstract

The induction of nerve injury response genes in Schwann cells depends on both transcriptional and epigenomic reprogramming. The nerve injury response program is regulated by the repressive histone mark H3K27 trimethylation (H3K27me3), deposited by Polycomb repressive complex 2 (PRC2). Loss of PRC2 function leads to early and augmented induction of the injury response gene network in peripheral nerves, suggesting H3K27 demethylases are required for derepression of Polycomb-regulated nerve injury genes. To determine the function of H3K27 demethylases in nerve injury, we generated Schwann cell–specific knockouts of H3K27 demethylase *Kdm6b* and double knockouts of *Kdm6b/Kdm6a* (encoding JMJD3 and UTX). We found that H3K27 demethylases are largely dispensable for Schwann cell development and myelination. In testing the function of H3K27 demethylases after injury, we found early induction of some nerve injury genes was diminished compared with control, but most injury genes were largely unaffected at 1 and 7 days post injury. Although it was proposed that H3K27 demethylases are required to activate expression of the cyclin-dependent kinase inhibitor *Cdkn2a* in response to injury, Schwann cell–specific deletion of H3K27 demethylases affected neither the expression of this gene nor Schwann cell proliferation after nerve injury. To further characterize the regulation of nerve injury response genes, we found that injury genes are associated with repressive histone H2AK119 ubiquitination catalyzed by PRC1, which declines after injury. Overall, our results indicate H3K27 demethylation is not required for induction of injury response genes and that other mechanisms likely are involved in activating Polycomb-repressed injury genes in peripheral nerve.

Myelination of peripheral nerve axons by Schwann cells has multiple important functions including trophic support, structural integrity, and enabling saltatory conduction (1, 2). These attributes allow robust transmission of action potentials and maintain axon homeostasis. However, the intrinsic plasticity of Schwann cells to promote repair of peripheral nerves involves several regenerative processes in response to Wallerian degeneration of axons, including both macrophage recruitment and clearance of myelin debris (3, 4, 5). Schwann cells reprogram themselves to support axon regeneration and undergo a transformation from a highly quiescent state to active repair cells that elongate to form Bungner bands that facilitate axon regeneration (6, 7, 8). These elaborate cellular behaviors do not depend on a stem cell niche (9) but rather reflect an innate ability of terminally differentiated Schwann cells to undergo a dramatic transdifferentiation to a more proliferative, proregenerative state called the repair Schwann cell (6).

The reprogramming is supported by an array of epigenetic changes converging into the overall response to simulate axon regeneration. After the nerve damage, substantial reshaping of the transcriptome occurs through dramatic changes in transcription factors as well as dynamic changes in acetylation and methylation of histones in Schwann cells (10, 11, 12, 13, 14). Among the most important transcription factors essential for nerve repair in Schwann cells is JUN, an AP-1 component that is required and sufficient for activation of many injury genes (8, 15). For many JUN target genes and other genes activated by injury, the basal levels of these aforementioned genes are low or absent in mature Schwann cells prior to injury due to repressive H3K27me3 made by the Polycomb repressive complex 2 (PRC2) (8, 12, 13, 14, 16). For instance, glial derived neurotrophic factor (*Gdnf*) is one of the PRC2-regulated genes important for nerve regeneration (17). Sonic hedgehog (*Shh*) is another highly induced gene that promotes regeneration (18). In our previous studies, we found that derepression of many nerve injury genes is accompanied by H3K27 demethylation after injury (12, 13, 14).

Canonical PRC2 is composed of core subunits including the lysine methyltransferases EZH1/2, suppressor of zeste 12 (SUZ12), and embryonic ectoderm development (EED) (19, 20), and there are a number of accessory subunits that play important roles. A Schwann cell–specific *Eed* conditional knockout mouse model exhibited premature induction of nerve injury genes in uninjured nerves (12). Moreover, the nerve injury experiment showed that *Eed* cKO mice had premature and/or augmented induction of nerve injury genes after injury (13), demonstrating the importance of H3K27me3 in repression of the injury-related program. In line with these findings, chromatin immunoprecipitation (ChIP) studies indicated that the promoters and gene bodies of many nerve injury genes in Schwann cells showed loss of H3K27me3 after injury (12), indicating that this histone mark acts as a switch for transcriptional induction.

Given that the Schwann cell responses to nerve injury are accompanied by H3K27me3 reprogramming, this provides a novel system to test the requirements for activation of PRC2 repressed gene networks that support axonal regeneration and proliferation. We hypothesized that active removal of H3K27me3 by H3K27 demethylases JMJD3/KDM6B and UTX/KDM6A is required for induction of the nerve injury network in Schwann cells after injury. JMJD3 and UTX proteins belong to the Jumonji family containing the catalytic JMJC protein demethylase domain (21). These H3K27 demethylases have been shown to be required for activation of Polycomb-repressed genes in other systems, including neural progenitor cell development and T cell differentiation (22, 23, 24). However, other demethylase-independent mechanisms for derepression have been characterized (25) and PRC2 repression is linked with PRC1 repression, which involves monoubiquitination of H2A (H2AK119ub1) (19). Therefore, we have also more fully characterized the repressed state of nerve injury genes by profiling H2AK119ub1 made by Polycomb repressive complex 1 (PRC1).

Previous studies suggested that JMJD3-mediated demethylation of H3K27 limits Schwann cell proliferation after injury by activation of the *Cdkn2a* gene (26). *Cdkn2a* encodes both INK4A/p16, which is a cyclin-dependent kinase inhibitor and tumor suppressor, and p19/ARF, an important regulator of p53 activation. Consistent with this model, the Schwann cell–specific knockout of *Eed* led to persistent *Cdkn2a* expression and a failure of Schwann cells to proliferate after injury (13). Of interest, studies showed that neurofibromas caused by *NF1* mutation most often transition to malignant peripheral nerve sheath tumor through the co-mutation of PRC2 subunit genes and *CDKN2A* (27, 28, 29). Therefore, we sought to test the hypothesis that *Jmjd3/Kdm6b* and *Utx/Kdm6a* are required to activate *Cdkn2a* (p16 and p19) and other injury genes after injury.

## Results

### Conditional inactivation of *Jmjd3* and *Utx* in Schwann cells

**Figure 1:**
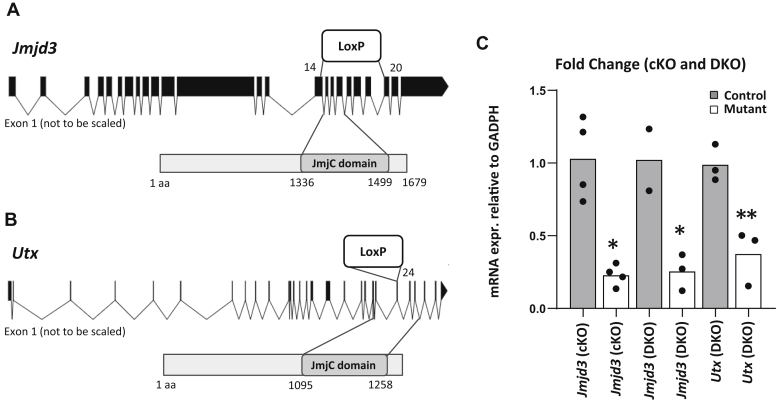
**Schwann cell–specific knockout of H3K27 demethylases.***A*, in the *Jmjd3* locus, the catalytic domain in exons 14 to 20 is flanked by loxP sites. *B*, similarly, the UTX locus contains the floxed 24th exon that would impair the catalytic function upon excision. *C*, qRT-PCR analysis of RNA extraction from distal stumps of control and DKO sciatic nerves 1 day after cut was performed. N = 4 for control and n = 4 for single *Jmjd3* cKO. n = 2 for control and n = 3 for DKO nerves. Data: mean ± SD; ∗∗*p* < 0.005, ∗*p* < 0.05 (one-way ANOVA).

To test the involvement of H3K27 demethylases in activation of the Schwann cell injury program, we made a conditional deletion of *Jmjd3/Kd*m6b specifically in Schwann cells using the *Mpz*-cre driver (30). The conditional allele (*Jmjd3* f/f) has exons 14 to 20 of the *Jmjd3* gene flanked by loxP sites (31) (Fig. 1*A*). This would result in deletion of the catalytic Jumonji domain, at E13.5 to 14.5 stage in Schwann cells, causing a frameshift in the C terminus of the protein. We validated the knockout of *Jmjd3* gene using quantitative RT-PCR with primers located within the deleted exons. The results showed ~76% loss in *Jmjd3* expression in mutant intact nerves compared with control intact nerves (Fig. 1*C*), which corresponds to the proportion of Schwann cells in peripheral nerve (12).

In order to test for potential redundancy in H3K27 demethylation, we also knocked out the other major H3K27 demethylase, *Utx/Kdm6a*, which is also expressed in RNA-Seq profiles of peripheral nerve (13, 32, 33). *Utx* is an X-linked gene composed of 29 exons (34), and the conditional allele contains loxP sites surrounding exon 24 coding for the catalytic domain of *Utx* (35) (Fig. 1*B*). After breeding this allele to the Mpz-cre driver line, we found a similar loss of ~70% of *Utx* expression in peripheral nerve from single and double knockouts (Fig. 1*C*). As seen in previous studies using Mpz-cre (14), the residual *Jmjd3* and *Utx* expression is likely due to the presence of non-Schwann cell types in peripheral nerve.

### Developmental effects of inactivating H3K27 demethylases

Before testing the involvement of *Jmjd3* and *Utx* in nerve injury, we evaluated whether deletion of *Jmjd3* and *Utx* affects Schwann cell development. In our previous analysis of the *Eed* knockout, peripheral nerve development was relatively normal up to 2 months of age, with development of hypermyelination and Remak bundle fragmentation at later time points (14). However, in the *Jmjd3/Utx* conditional knockouts, there are no obvious behavioral phenotypes typically found in peripheral neuropathy such as hindlimb clasping even up to 7 months of age, and visual inspection showed the normal opaque appearance of myelinated nerve. Despite the apparent lack of such behavioral phenotype, we examined nerve ultrastructure by performing electron microscopy analysis of sciatic nerve in the Schwann cell–specific double knockout of *Jmjd3* and *Utx*.

**Figure 2:**
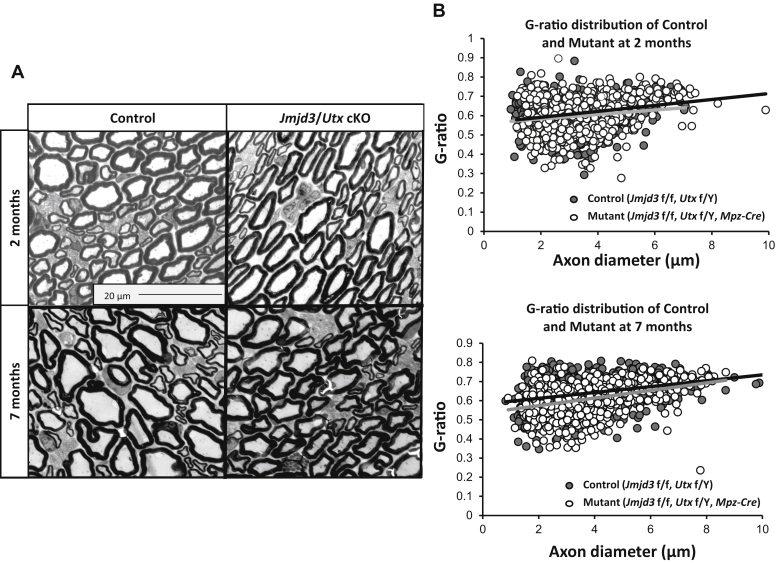
**Schwann cell H3K27 demethylase activity is not required for development.***A*, electron micrographs of the sciatic nerves at the indicated developmental time points of nerves of *Jmjd3/Utx* DKO mice and littermate controls. Scale bar represents 20 μm. *B*, for g-ratio analysis (axon diameter/diameter of myelinated fiber), the diameter of axon and outer diameter of myelinated fiber were measured on over 900 randomly selected fibers per genotype. Data: n = 3 per genotype and age.

The electron microscopy images of mature myelin in sciatic nerves of ~2-month-old mutant mice showed a normal distribution of axon diameters and myelin sheaths compared with controls (Fig. 2*A*). Myelin thickness was measured using the g-ratio, which is axon diameter divided by outer diameter of myelin sheath, which is plotted against the axon diameter. The linear regressions of both distribution plots for both control and mutant mice are virtually aligned, indicating no significant difference in myelin thickness compared with control (Fig. 2*B*). The number of myelinated fibers is also comparable in both genotypes.

Our previous analysis of the *Eed* knockout identified progressive defects in hypermyelination and Remak bundle integrity at later time points (14). Therefore, we also harvested the sciatic nerves of 7-month-old double knockout mice and processed for electron microscopy imaging. The morphology is similar between mutant and control genotypes as no deformities were observed in the mutant nerve (Fig. 2*A*). Abnormalities like myelin outfolding and development of tomacula caused by excess myelin membranes were observed in the *Eed* knockout (14). However, no myelin pathology is seen in Schwann cell–specific knockout of the two H3K27 demethylases. Although we had observed some difference in Remak bundles in *Eed* cKO mice (14), they appear to be intact in mutants, similar to those of controls and no major deformity is consistently observed in the double knockout nerves. Therefore, H3K27 demethylase activity is not required for myelin development or maintenance.

### Control of proliferation after injury

**Figure 3:**
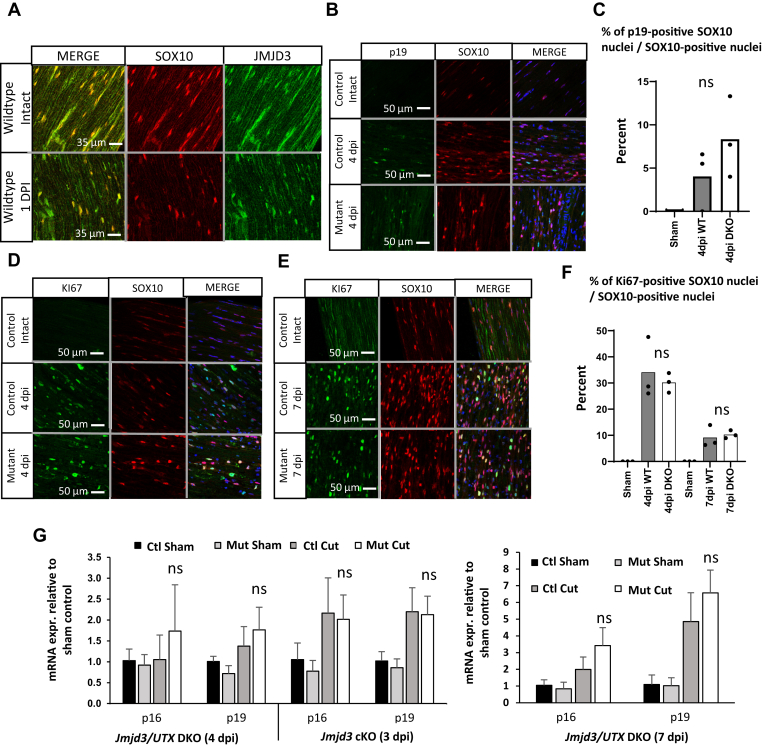
**H3K27 demethylase activity is dispensable for Schwann cell proliferation after nerve injury.***A*, immunofluorescence analysis of the longitudinal sections from distal stumps of wildtype sciatic nerves 1 day post injury (dpi) was performed using the indicated antibodies. n = 3 for control and n = 3 for 1 dpi nerves. *B*, immunofluorescence analysis to measure p19, a cell cycle inhibitor, was performed in the sections of control and DKO sciatic nerves at 4 days after crush. *C*, the quantification of p19 at 4 dpi. *D* and *E*, immunofluorescence analysis to measure Ki67, a cell proliferation marker, was performed in the sections of control and DKO sciatic nerves at various time points after injury utilizing both transection and crush. *F*, the quantification of Ki67. *G*, qRT-PCR analysis was used to identify the expression level of p19 and p16 transcripts of *Cdkn2a* from *Jmjd3* cKO and DKO and control sciatic nerves of uninjured condition or 3 days after cut/4 days after transection/crush or 7 days after cut. Expression levels were normalized with *Gapdh*. Data: mean ± SD; n = 6 for sham and n = 6 for 3 dpi *Jmjd3* cKO, n = 4 for sham and n = 4 for 4 dpi DKO, and n = 3 control and n = 5 for 7 days postcut DKO (one-way ANOVA). Ns, not significant

It had been reported that JMJD3 is induced after injury (26), which was proposed to regulate Schwann cell proliferation after injury by removing H3K27 methylation from the *Cdkn2a* gene that encodes both p16 and p19 tumor suppressor proteins. However, examination of nerve injury RNA-Seq datasets did not show that *Jmjd3* is induced at the transcript level (13, 33). Although there could be posttranscriptional induction of JMJD3, we found that JMJD3 protein is expressed even before injury in Schwann cells in contrast to the earlier findings (Fig. 3*A*). We tested the specificity of our antibody using siRNA for *Jmjd3* in the cultured S16 cells (Fig. S1). In addition, the protein level of JMJD3 did not appear to significantly increase after nerve injury, as measured by the immunofluorescence and Western blot. Nonetheless, JMJD3 could regulate nerve injury gene induction at 1 day post injury (dpi) even in the absence of induced protein levels.

The normal induction of *Cdkn2a* (p16 and p19 transcripts) occurs at 3 to 7 days after injury, and earlier data suggested that JMJD3 would be required for this induction (26). In partial support of this model, we had previously investigated the effect of H3K27me3 depletion in Schwann cell–specific *Eed*-cKO mice and found constitutively high *Cdkn2a* transcript expression (p19 and p16) along with reduced proliferation of Schwann cells after injury (13). Therefore, this model would predict that loss of H3K27 demethylases in Schwann cells would result in increased proliferation owing to the inability to derepress *Cdkn2a* after injury. To test if *Jmjd3* is required for *Cdkn2a* induction and regulation of cell proliferation after peripheral nerve injury, we performed immunofluorescence for p19 and Ki67 at 4 days after nerve crush. We quantitated the number of cells positive for p19 and Ki67 along with Schwann cell marker SOX10. In the *Jmjd3/Utx* double knockout, we found that there was no difference from control at the 4-dpi time point for both p19 induction and proliferation (Fig. 3, *B* and *C*). Since proliferation normally subsides by 14 days after injury, it is possible that the proliferation would be maintained longer in the absence of *Jmjd3* and *Cdkn2a* induction. However, the level of proliferation at longer time points after injury, at both 7 and 14 dpi, was not significantly different from control (Fig. 3, *D*–*F* and Fig. S2). In addition, we also determined the transcript levels of p16 and p19 transcripts of *Cdkn2a* using qRT-PCR at 3/4 and 7 dpi. We found no significant difference between genotypes (Fig. 3*G*). Therefore, neither *Jmjd3* nor *Utx* is required for *Cdkn2a* induction, and their deletion has no major effects on proliferation after injury.

### H3K27 demethylases are not required for macrophage infiltration after nerve injury

**Figure 4:**
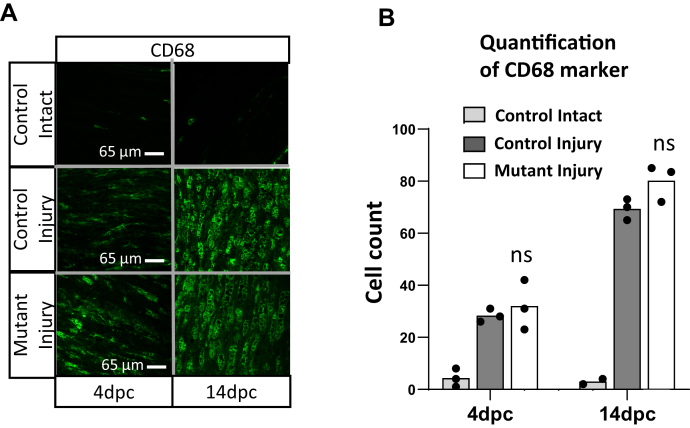
**H3K27 demethylase activity is not required for macrophage recruitment after nerve injury.***A*, immunofluorescence analysis of the longitudinal sections from distal stumps of control and *Jmjd3/Utx* cKO sciatic nerves at various time points after crush was performed using the indicated antibodies. *B*, quantification of CD68. Data: n = 3 for control and n = 3 for DKO nerves at 4 dpc and n = 2 for control and n = 3 for 14 dpc.

Another major component of the nerve injury response involves Schwann cell production of chemokines (*e.g.*, *Mcp1*/*Ccl2*) that recruit macrophages that clear out the myelin debris that can inhibit axonal regeneration (36, 37, 38, 39). To test whether the demethylase knockout may have effects on the activities of immune cells, we examined the CD68 macrophage marker by immunofluorescence to assess macrophage infiltration and observed no significant difference between genotypes at 4 and 14 dpi (Fig. 4, *A* and *B*), indicating that demethylases are not required for macrophage recruitment by Schwann cells in injured nerve.

### Regulation of nerve injury genes by H3K27 demethylases

**Figure 5:**
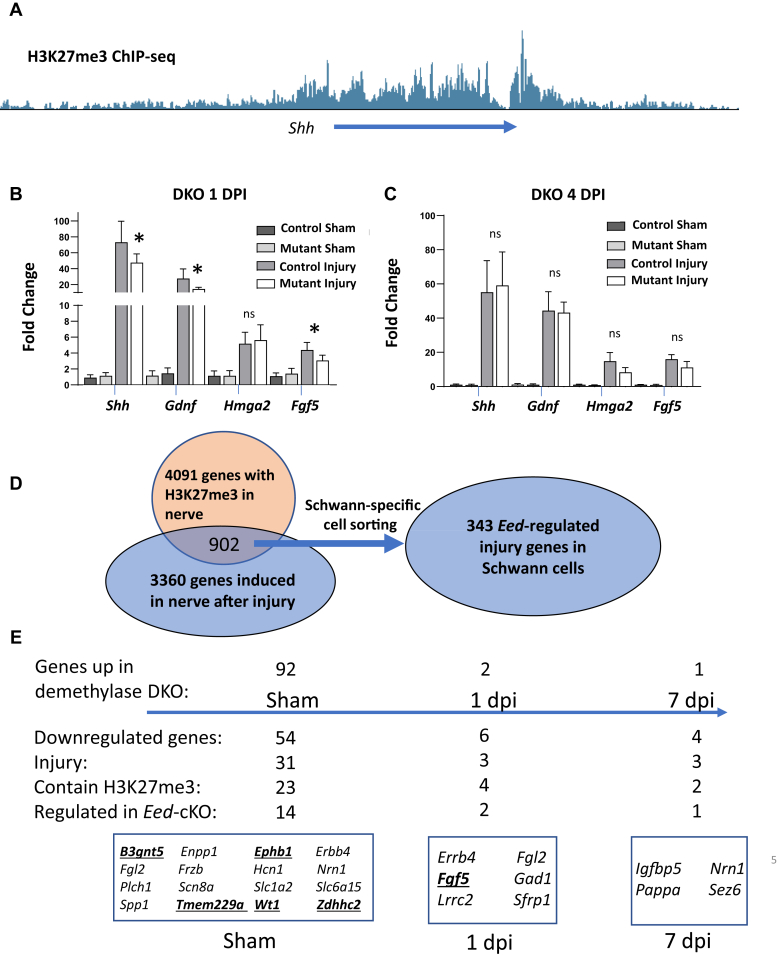
**H3K27 demethylase activity is not absolutely required for the induction of nerve injury genes.***A*, ChIP track of Sonic hedgehog (*Shh*) locus shows the association of repressive H3K27me3. *B*, qRT-PCR analysis was used to identify the expression level of injury-responsive genes from 2-month *Jmjd3* cKO and control sciatic nerves in uninjured condition or 1 day after transection. Expression levels were normalized with *Gapdh*. Data: mean ± SD; *asterisks* indicate *p*-value between genotypes in the respective condition. ∗*p* < 0.05; n = 5 for control and n = 7 for DKO (one-way ANOVA). *C*, qRT-PCR analysis displays the expression of indicated nerve injury genes in distal stumps of nerves, indicated genotypes 4 days after crush. Data: mean ± SD; *asterisks* indicate *p*-value between genotypes in the respective condition. ∗*p* < 0.05; n = 6 for control and n = 6 for DKO (one-way ANOVA). *D*, the Venn diagram shows intersection of gene sets with H3K27me3 and known injury genes. The list of 4091 genes with H3K27me3 was filtered by >10 peak score (13). The combined 1 and 7 dpi list of 3360 unique injury genes was obtained from the RNA-Seq datasets (13, 32), filtered by <0.05 *p*-value and >2-fold change. The gene list was further filtered for Schwann cell–specific expression using published cell sorting data (33). *E*, tables summarize and highlight the downregulated genes from DKO samples compared with control with significant *p*-values across sham and 1, and 7 days after injury.

We had found that a significant subset of nerve injury genes is regulated by PRC2, including *Shh* (Fig. 5*A*), *Gdnf*, and *Runx2* (12, 13, 14). Several of these genes are rapidly induced after injury within 24 h, whereas the levels of other injury-induced transcripts become induced at later time points (3–7 days) (13). Therefore, we hypothesized that the *Jmjd3* and *Utx* knockouts would block the transcriptional induction of many of these genes after nerve injury. We utilized quantitative RT-PCR analysis of sciatic nerve RNA in sham and injured nerve from both the single knockout of *Jmjd3* and double knockout of *Jmjd3/Utx* mouse lines to measure the injury induction of selected PRC2-repressed genes. Although there are other cell types in nerve, the downstream response of nerve injury genes like *Shh* and *Gdnf* is specific to Schwann cells (8, 33). In the *Jmjd3/Utx* conditional knockout, qRT-PCR experiments with 1 and 4 dpi RNA samples of DKO nerve were conducted (Fig. 5, *B* and *C*). We observed that the induction of *Shh*, *Gdnf*, and *Fgf5* was modestly impacted in double knockouts at 1 dpi compared with control. However, the induction of these genes recovered to normal injury-induced levels at 4 dpi.

We observed no significant changes in the induction of several nerve injury genes such as *Shh*, *Gdnf*, *Fgf5*, *Runx2*, and *Hmga2* in the *Jmjd3* knockout at 1 dpi (Fig. S3*A*). In contrast, the induction of *Fgf5* and *Hmga2* was slightly reduced at 3 dpi (Fig. S3*B*). At 7 dpi in *Jmjd3* cKO, all of the genes with the exception of *Fgf5* recovered to the levels observed in the wildtype mice (Fig. S3*C*).

We assessed H3K27 demethylase dependence of global gene expression by performing RNA-Seq at 1 and 7 days post nerve transection. We identified differentially expressed genes by comparing two datasets (1 dpi DKO *versus* 1 dpi control, 7 dpi DKO *versus* 7 dpi control). After injury, there were only a few genes that were significantly lower in the DKO (<0.05 *p*-value, FDR corrected). Several studies have characterized the nerve injury program in Schwann cells using RNA-Seq (13, 32, 33), and ChIP-seq analysis identified 4091 genes associated with H3K27me3 in intact nerves (12, 13, 14). Using these datasets, we had identified injury-induced genes that are associated with H3K27me3 resulting in 902 genes and refined them using RNA-Seq analysis of sorted Schwann cells after nerve injury (33) to ensure that their induction occurs in Schwann cells. Of these, there were 343 injury-induced genes that were found to be regulated by PRC2, since they were induced in the *Eed* cKO before and/or after injury (Fig. 5*D*).

At 1 dpi, there are only six genes that are significantly lower in the double knockout (Fig. 5*E*), and three of them are previously defined as PRC2-regulated injury genes (*Fgf5*, *Sfrp1*, *Erbb4*). *Fgf5* is a growth factor that can stimulate axon growth and is induced after injury in Schwann cells (36). *Erbb4* belongs to a family of important ErbB receptor family mediates neuregulin-1 signaling. Similarly, at 7 dpi, there are only four genes that are significantly lower. One of them had also been defined as PRC2-regulated injury gene: *Igfbp5*, which is a modulator of IGF signaling and has previously been characterized to have a role in peripheral axon regeneration (40, 41), and two others (*Pappa* and *Sez6*) are injury-induced genes associated with H3K27me3. Therefore, the demethylase activity of JMJD3 and UTX appears to be largely dispensable for the induction of the injury program except for a few relevant injury genes (*Fgf5*, *Sfrp1*, *Erbb4*).

Although relatively few injury genes are affected by loss of H3K27 demethylase activity, we identified 146 genes that are significantly altered in the DKO prior to injury (sham). Of the 54 genes that are downregulated, 23 genes are associated with H3K27me3. This analysis highlighted a potential discrepancy with the injury time points, where much fewer genes were lower in the DKO. However, it turns out that 31 of these 54 genes are regulated by injury, and most are significantly downregulated after injury, which explains why most of these are not differentially regulated in the DKO after injury. Since they are downregulated after injury, they are associated with the myelination program in Schwann cells, and their decrease in the DKO indicates that demethylases are required for their developmental induction in Schwann cells. Many of the genes induced during Schwann cell myelination are dependent on the EGR2 transcription factor (42, 43), and several of the downregulated genes had been identified as EGR2 target genes: *Frzb*, *Hcn1 Fgl2*, *Slc6a15*, *Spp1*. However, most of the EGR2 regulatory network including major myelin genes (43) was unchanged, consistent with the normal nerve morphology described above.

Overall, both RNA sequencing datasets showed that a majority of PRC2-regulated genes (13) remained unchanged in double cKO nerves under both cut and sham conditions (Fig. S4, *A* and *B*). In addition, the lack of any morphological defects and the small number of deregulated genes suggest that demethylases are not essential for Schwann cell development, although their absence leads to a delayed induction of some injury genes.

### Histone modification changes in nerve injury genes

In previous studies, we had found that the levels of H3K27me3 at the promoters of nerve injury genes such as *Shh* and *Gdnf* decreased within 24 h after peripheral nerve injury. To test that the H3K27me3 mark at such sites is actually being targeted by demethylase, we utilized the Schwann cell–specific knockout of *Jmjd3* and performed ChIP in sham and cut nerves with H3K27me3 antibody. In contrast to the previously described reduction of H3K27me3 levels after injury, the level of H3K27me3 in several injury gene promoters remained elevated after injury in the *Jmjd3* cKO, indicating that the injury-induced reduction of H3K27me3 is *Jmjd3* dependent (Fig. S5).

**Figure 6:**
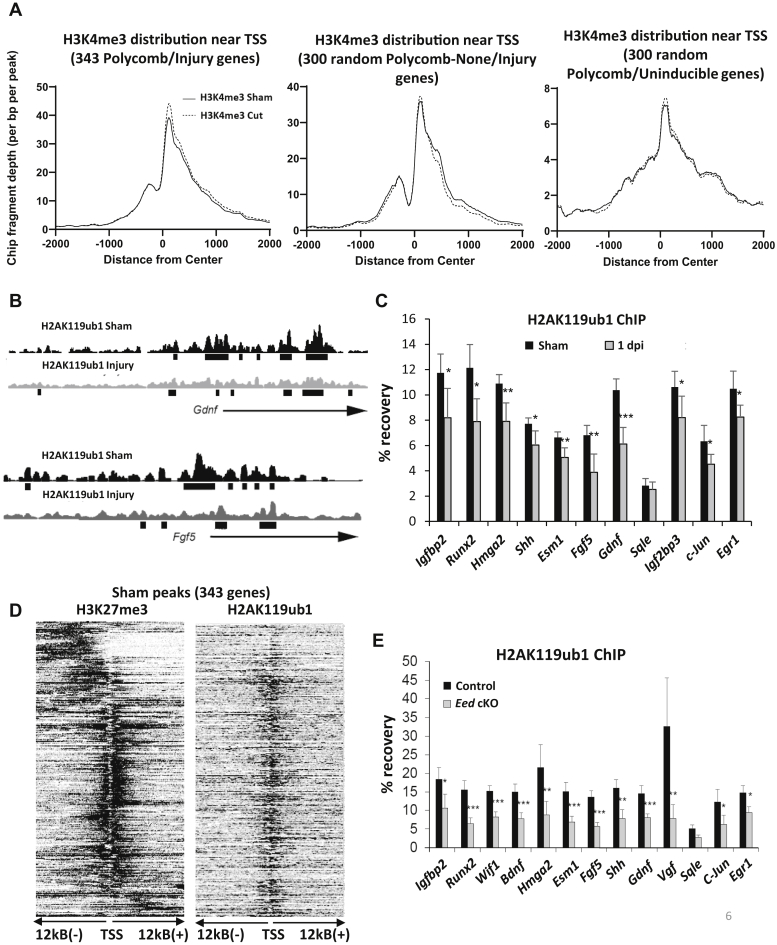
**H2AK119ub1 is dynamically regulated during injury gene induction.***A*, the average distribution plots of histone mark H3K4me3 at TSS were based on the respective lists of Polycomb-regulated genes, 300 randomly selected injury genes lacking H3K27me3, and 300 randomly selected uninduced Polycomb genes after injury. The H3K4me3 ChIP-seq data were generated using two replicates. *B*, ChIP-seq tracks of Growth-derived neural factor (*Gdnf*) and Fibroblast growth factor 5 (F*gf5*) loci show the association of repressive H2AK119ub1 mark at the promoter sites and the loss of such mark after injury. *C*, ChIP analysis of lysates from distal stumps of control and wildtype sciatic nerves 1 day after cut was performed using the H2AK119ub1 antibody. n = 5 for control and n = 5 for cut nerves. Data: mean ± SD; ∗∗*p* < 0.005, ∗*p* < 0.05 (one-way ANOVA). *D*, the heatmaps display the distribution of H3K27me3 and H2AK119ub1 centered at TSS based on 343 Polycomb-regulated gene list. The H3K27me3 and H2AK119ub1 ChIP-seq data were generated using two replicates. *E*, ChIP analysis of lysates from intact control and *Eed* cKO sciatic nerves was performed using the H2AK119ub1 antibody. n = 5 for control and n = 5 for *Eed* cKO nerves. Data: mean ± SD; ∗∗∗*p* < 0.0005, ∗∗*p* < 0.005, ∗*p* < 0.05 (one-way ANOVA).

Although H3K27me3 had been implicated as the bottleneck to the activation of injury gene network in previous reports (12, 13, 14, 26), there are demethylation-independent mechanisms that could overcome Polycomb repression after nerve injury (19, 25). It is possible that increased levels of H3K4me3 alone could be sufficient to activate Polycomb-repressed genes. We had previously found that H3K4me3 marks are associated with promoters of *Shh*, *Gdnf*, *Hmga2*, and other PRC2-regulated genes (12), which indicates that these promoters correspond to the previously defined bivalent state of promoters associated with both H3K27me3 and H3K4me3 (44, 45). Using our H3K4me3 ChIP-seq data, we examined distribution of H3K4me3 marks on H3K27me3-associated injury genes in peripheral nerve in intact and injured conditions. A distribution plot of H3K4me3 on the 343 H3K27me3-associated injury genes shows a narrow peak at the TSS that increases at 1 day post injury compared with that of intact condition (Fig. 6*A*). The increased H3K4me3 is a plausible explanation for some of the most dramatic early changes in injury gene activation, which can occur several days in advance of detectable increases in transcript levels. As controls, we observed no significant changes in the level of H3K4me3 for randomly selected 300 injury genes lacking H3K27me3 and another 300 randomly selected uninduced H3K27me3-associated genes after injury.

### Injury-induced depletion of H2A ubiquitination

Studies of Polycomb repression have elucidated an important role of the PRC1 complex that modifies H2AK119 by ubiquitination. The traditional model has been that Polycomb repression occurs sequentially where H3K27me3 is first deposited to the site of gene loci and then attracts Polycomb repressive complex 1 that in turn deposits the H2A119ub1 to further repress the gene activity (19). However, although H3K27 methylation does often overlap with H2AK119ub1, H2A ubiquitination can affect PRC2 recruitment and activity (46), and it has been shown that these modifications can be established and regulated independently of each other (19, 47).

This raises the possibility that H2AK119ub1by PRC1 is involved in the repression of nerve injury genes and furthermore that deubiquitination of this histone mark is required for their induction (48, 49). We therefore performed the nerve injury experiment followed by ChIP using an H2AK119ub1 antibody. We found that many of the previously defined PRC2-repressed injury genes are associated with H2AK119ub1 at the promoter sites, which is lost after injury (Fig. 6, *B* and *C*). We also performed ChIP-seq for H2AK119ub1 in sham and injured nerve. In sham nerve, we found a large overlap of H2AK119ub1 with the H3K27me3 on injury-induced promoters (Fig. 6*D*). We plotted the average distribution of H2AK119ub1 at the promoters of 343 Polycomb regulated genes and found significant enrichment flanking the transcription start sites. In addition, the peak distribution is lower after injury compared with sham (Fig. S6). This can be seen for two individual genes (*Gdnf* and *Fgf5*), which show changes in their H2AK119ub1 profiles in sham *versus* injured nerve.

Our results suggest a new model in which H2A deubiquitination would play a key role in reversing Polycomb repression rather than H3K27 demethylation. Therefore, our previous observations of injury gene derepression in the *Eed* cKO would predict that loss of *Eed* may trigger loss of H2A ubiquitination. We tested that by performing H2Aub1 ChIP assays in the *Eed* cKO, and we did indeed find a significant loss of H2AK119ub1 in uninjured nerve of the *Eed* cKO (Fig. 6*E*). Therefore, our data indicate that both H2A ubiquitination and H3K27 demethylation may be required for proper regulation of injury genes.

## Discussion

Many nerve injury genes are repressed by H3K27me3, and their expression remains low or absent in mature peripheral nerve. Previous studies had suggested that demethylation is required for induction of nerve injury and cell cycle genes such as *Shh*, *Gdnf*, and *Cdkn2a*, promoting the regeneration program to proceed in an appropriate and timely manner (26). This idea was supported by previous studies of a Schwann cell–specific deletion of *Eed* (12, 13, 14), showing that preventing PRC2 repression can exert significant effects on the injury program. Therefore, we determined whether demethylases are required for gene activation after injury.

In development of Schwann cell–specific knockout of H3K27 demethylases, we first examined if they were required for Schwann cell development before assessing their roles in the nerve injury response. The H3K27 demethylase activity of JMJD3 and UTX is not essential for Schwann cell development and myelination. No abnormal developmental phenotype is seen at the maintenance stage of Schwann cells and the time point beyond 2 months of age in the mouse models. Therefore, we could focus on the early gene induction events in the aftermath of injury. Our primary hypothesis was that PRC2-regulated genes required H3K27 demethylases to be induced in the early phase of regeneration, many of which are induced 24 h after injury. Any epigenetic changes required for induction should occur at this early time point, and we had previously observed decreased H3K27 methylation at this time (12, 13, 14). However, for most injury genes, the demethylase activity of JMJD3 and UTX are not required for their induction with the exception of a few genes like Fgf5. Some injury genes were still lower at 4 dpi, but RNA-Seq from DKO 7 dpi nerves showed that there were only four significantly altered genes across the injury-induced transcriptome. Given the relatively subtle effects of nerve injury gene induction and their recovery by 7 days, it is not likely that extended analysis of nerve regeneration will reveal a phenotype following the nerve injury in the DKO.

It had been reported that JMJD3 is induced in mouse model after 5 days post nerve transection by immunofluorescence and had proposed that JMJD3 has a significant role in Schwann cell proliferation after injury through demethylation of the *Cdkn2a* gene (26). Our earlier studies had confirmed the presence of H3K27me3 on the *Cdkn2a* promoters, and we also had observed overexpression of both p19 and p16 transcripts and reduced proliferation after injury with a Schwann cell–specific knockout of *Eed* (13). The regulation of *Cdkn2a* gene by PRC2 has relevance to the Schwann cell–derived tumors in neurofibromatosis caused by mutation of the *NF1* tumor suppressor gene (50), The progression from neurofibromas in *NF1* to the more malignant form called malignant peripheral nerve sheath tumor is often accompanied by comutation of the *CDKN2A* gene and genes encoding subunits of the PRC2 complex (*e.g.*, *EED* and *SUZ12*) (27, 28, 29). Indeed, *NF1* microdeletions are more predisposed to malignant peripheral nerve sheath tumor owing to the deletion of both *NF1* and the neighboring *SUZ12* gene (51).

We tested the proliferation in double knockout of demethylases expecting there would be increased and/or prolonged Schwann cell proliferation after injury if H3K27 demethylases were required for induction of p19 and p16. However, despite the evidence for PRC2 regulation of *Cdkn2a* and Schwann cell proliferation after injury, our double knockout results showed that H3K27 demethylases are not required for *Cdkn2a* induction or regulation of Schwann cell proliferation after injury. There is no significant difference between genotypes in terms of proliferation at 7 and 14 dpi. In addition, our immunofluorescence data using independent antibodies for JMJD3 indicate that it is expressed in Schwann cells prior to nerve injury. Given the early changes in H3K27me3 at 1 day post injury, this is likely due to targeting/activation of pre-existing JMJD3 protein.

Although previous studies have shown that demethylase activity regulates the H3K27 methylation status, it has been documented that JMJD3 and UTX have demethylation-independent activities and they are constituents of larger complexes with MLL proteins and also associated with BRG1-containing complexes (21). In the conditional mutants used here, the loxP sites remove exons containing the catalytic domain, with a resulting frameshift leading to loss of the entire C terminus. Nonetheless, JMJD3 and UTX could be involved in the injury gene regulation in a demethylase-independent manner. The previously described association of H3K27 demethylases with MLL complexes (21) is consistent with the increased H3K4me3 at injury genes (12).

Most of the genes that were found to be JMJD3/UTX dependent were found in the sham condition. This gene set was much larger than that found in the 1 and 7 dpi RNA-Seq datasets, but most of the downregulated genes are ones that naturally decrease after nerve injury based on several datasets (13, 32, 33), which explains why they were not significantly different after injury in the DKO. Their decrease after injury implies that they are coregulated with the rest of the myelination program that is dependent on Schwann cell contact with axons. Indeed, several of this set are among the genes regulated by the promyelinating EGR2/KROX20 transcription factor (43). In turn, this suggests that H3K27 demethylase activity is required for full induction of a subset of the myelin program. However, we did not detect any overt myelination defects, and many of these genes are decreased in the range of 40% to 75%. Evaluation of the entire EGR2-regulated gene network shows that most genes are unchanged in the DKO. Nonetheless, many of these genes are associated with H3K27me3. We speculate that genes that increase in the sham DKO samples are mechanistically linked to the downregulated genes, perhaps involving a transcriptional repressor such as WT1.

There are several possible mechanisms by which Polycomb repression can be bypassed without demethylation. For example, the proliferation of Schwann cells after injury, typically beginning at 3 to 4 days after injury, can lead to the passive reduction of H3K27me3 marks if H3K27 methylation is not maintained after DNA replication (52). This could explain the lack of effect at 7 days post injury but likely does not explain the early induction of injury genes at 1 day given the time course of Schwann cell division after injury. A second potential model is the H3K4me3 histone mark, which is commonly associated with active promoters. Many nerve injury genes are associated with bivalent modification of H3K27me3 and H3K4me3 (12), and increased H3K4me3 is consistent with the traditional framework of activation through rebalancing of Polycomb and Trithorax-like mechanisms (44, 45, 53). Therefore, mechanisms to increase H3K4 methylation could be sufficient to activate Polycomb-repressed injury genes. Finally, many Polycomb-repressed genes contain both H3K27me3 and H2AK119ub1 formed by PRC1. PRC1 modifications were thought to be entirely dependent on H3K27 methylation, but more recent studies indicate that PRC1 and PRC2 can be regulated independently and that PRC1 activity may stimulate recruitment of PRC2 (19, 54). Using ChIP-seq analysis, we found a general colocalization of H3K27me3 and H2AK119ub1 consistent with previous studies, but some injury genes only have evidence of PRC1 repression. However, the ChIP-seq data suggest a fairly significant decrease of H2AK119ub1 at 1 day after injury. Therefore, it may be that antagonizing PRC1 repression is the primary means of activation, and removal of H3K27me3 may simply be a consequence of H2A deubiquitination. Several different H2A deubiquitinases have been identified, which could play a role in injury gene activation, including MYSM, USP16, and BAP1 (49, 55).

The activation of the nerve injury program is regulated by transcription factors like JUN, but coordinate activation of this regenerative program requires definition of the mechanisms by which the nerve injury program is repressed. Some nerve injury genes are active in early neural crest and Schwann cell development (12) and become repressed in differentiated Schwann cells, and others (like Shh) appear to be induced *de novo* without having been expressed in their differentiation from neural crest (16). The repressed status of nerve injury genes involves not only Polycomb repression but also repression by transcription factors like *Zeb2* (56, 57) and epigenomic remodelers such as histone deacetylases and the NuRD complex (10, 58, 59). We have not observed derepression of all H3K27me3-associated genes in the *Eed* cKO (13), so it is possible that derepression will involve multiple epigenomic complexes to maintain repression of the nerve injury program while allowing for its rapid induction after injury. Our studies highlight for the first time the involvement of PRC1 repression in this program, which is the focus of ongoing studies.

## Experimental procedures

Information on primer sequences and antibodies used in the paper are provided in Table 1.

**Table 1:** qRT-PCR primers (mouse)

*Runx2*	Forward	ACCAAGTAGCCAGGTTCAAC
	Reverse	GAGGATTTGTGAAGACTGTTATGG
*Fgf5*	Forward	AAAAGCCACCGGTGAAACC
	Reverse	TCACTGGGCTGGGACTTCTG
*Shh*	Forward	CAGCGACTTCCTCACCTTCCT
	Reverse	AGCGTCTCGATCACGTAGAAGAC
*Gdnf*	Forward	TCTCGAGCAGGTTCGAATGG
	Reverse	AAGAACCGTCGCAAACTTTACC
*Hmga2*	Forward	CAAGAGGCAGACCTAGGAAAT
	Reverse	CTCTTGCGAGGATGTCTCTTC
*Cdkn2a/p16*	Forward	GAATCTCCGCGAGGAAAGC
	Reverse	TGTCTGCAGCGGACTCCAT
*Cdkn2a/p19*	Forward	CACCGGAATCCTGGACCAGG
	Reverse	CACCGTAGTTGAGCAGAAGAGCT
*Kdm6b/Jmjd3*	Forward	CATGAACACCGTGCAGCTAT
	Reverse	CTCATGTACCGCGAACCACT
*Kdm6a/Utx*	Forward	AATATTGGCCCAGGTGACTG
	Reverse	TCACAGAAGTCATTCAAAACACC
ChIP primers		
*Shh* +3307	Forward	GGAAGCGCAGACAGACACTCT
	Reverse	CACAACAGCCTGGCACTCTCT
*Gdnf*	Forward	CCCCTGGATTGCGTGCTC
	Reverse	GGACATTAACTCCAAGTGGCCC

Antibodies	Catalog number	Company
SOX10	AF2864	R & D systems
Ki67	Ab16667	Abcam
p19/ARF	Sc-32748	Santa Cruz
CD68	Ab125212	Abcam
JMJD3	#A9780	Abclonal
ACTB	#AC004	Abclonal
IgG	12-370	Upstate/Millipore
H2AK119ub1	8240	Cell Signaling Technology
H3K27me3	AM39155	Active motif

### Mouse nerve injury surgery

Animal experiments were performed according to protocols approved by the University of Wisconsin, Madison School of Veterinary Medicine. *Kdm6b/Jmjd3*-floxed mice (31) and *Kdm6a/Utx* floxed mice (Jax#024177) (35) were maintained on the C57BL/6 genetic background (24) and mated to mP0TOTA-Cre (*Mpz*-cre) (30). Double *Jmjd3/Utx* floxed mice were generated, and homozygous floxed *Jmjd3* and *Jmjd3/Utx* alleles with and without the Mpz-cre transgene were used for experiments. Prior to surgery, animals were anesthetized with isoflurane (Piramal Healthcare), and an injection of 5 mg/kg ketoprofen was given for analgesia. A 5-mm-long incision was made through the skin and muscle exposing the sciatic nerve. The nerve was either cut as close to the proximal lateral region of the femur as possible or crushed 1 min using fine forceps. As a control, the contralateral leg also received a sham operation consisting of only a skin and muscle incision. The skin wound was sutured with rodent surgical staples. Six wildtype and knockout nerve tissues distal to the transection or sham site were isolated with epineurium removed and frozen immediately in dry ice and stored at −80 °C for further processing in ChIP experiments.

### Chromatin immunoprecipitation

Six freshly dissected mouse sciatic nerves per condition were minced in 1% formaldehyde for 8 min and then quenched for 10 min with glycine to a final concentration of 0.125 M. Samples were sequentially lysed in buffers LB1, LB2, and LB3 + 0.03% SDS (11). DNA was fragmented to an average size of 0.5 to 2 kb using 5× for 10 min Bioruptor (Diagenode) cycles on the medium setting. Each aliquot of sonicated chromatin (150 μg) was incubated overnight at 4 °C with 5 μg of antibody. A 10% aliquot was saved for input analysis. An 80-μl aliquot of protein G Dynabead (Invitrogen) slurry was added to each ChIP sample, rotating overnight at 4 °C. Immunoprecipitations were washed three times in RIPA buffer and then eluted at 65 °C in reverse cross-linking buffer (50 mM Tris, 10 mM EDTA, 1% SDS). ChIP DNA was purified by phenol chloroform extraction and resuspended in 10 mM Tris, pH 8.0. Antibodies used in the study are normal rabbit IgG (Millipore, 12-370) and H3K27me3 (Active Motif, 39155). Statistics were calculated using Student's *t* test. Error bars represent standard deviation, and asterisks denote *p* value (∗*p* ≤ 0.05; ∗∗*p* ≤ 0.005). The samples were generated from independent chromatin pools (n = 3 for H3K27me3 ChIP) and were analyzed using quantitative PCR primers listed in (Table 1).

### Electron microscopy and morphometric quantification

Freshly dissected sciatic nerves were immersion fixed in a solution of 2.5% glutaraldehyde, 2.0% paraformaldehyde in 0.1 M sodium phosphate buffer, pH 7.4, overnight at 4 °C. The nerves were then postfixed in 1% osmium tetroxide in the same buffer for 2 h at room temperature. Following OsO_4_ postfixation, the nerves were dehydrated in a graded ethanol series and then further dehydrated in propylene oxide and embedded in Epon resin. Ultrathin transverse sections were contrasted with Reynolds lead citrate and 8% uranyl acetate in 50% ethanol. Images were obtained with a Philips CM120 electron microscope with an AMT BioSprint side-mounted digital camera at the UW Medical School Electron Microscope Facility. Densitometric quantification was performed using NIS-Elements 4.0. Three mice per genotype were analyzed, and statistical analyses were evaluated by one-way ANOVA in all the experiments.

### Immunofluorescence

Freshly dissected nerves were embedded in Tissue-Tek OCT compound (Sakura Finetek) and snap frozen with liquid nitrogen. Longitudinal or transverse cryostat sections (10 μm) were air dried for 5 min and fixed in 4% paraformaldehyde for 10 min. The sections were then blocked in PBS containing 5% donkey serum/1% BSA/0.5% Triton-X 100 for 1 h at room temperature. Primary antibody incubation was performed overnight at 4 °C in PBS containing 5% donkey serum/1% BSA/1% Triton-X 100, and secondary incubation was performed in PBS at room temperature for 1 h. Hoechst 33342 (1:5000 in PBS, Sigma) was applied to stain nuclei for 1 min. Three 4-min washes were performed in PBS after fixation and blocking, and in PBS containing 0.1% Tween20 after primary antibody incubation and nuclear staining. After coverslips were mounted using Fluoromount-G (SouthernBiotech), sections were examined on a Nikon A1R confocal and quantitated by both Columbus imaging software and manual curation.

### Western blot

Freshly dissected nerves were snap frozen with liquid nitrogen and crushed. The nerves were then homogenized in lysis buffer (50 mM Tris-HCl at pH 6.8, 10% glycerol, 2% SDS, 10% β-mercaptoethanol, 50 mM NaF, 1 mM Na_3_VO_4_ and Protease Inhibitor Cocktail [Sigma, P8340]) using a motorized pellet pestle. Cells in culture were homogenized in 3x lysis buffer. After a 15-min incubation in ice, lysates were boiled at 95 °C for 3 min and centrifuged at 4 °C for 15 min. Subsequently, supernatants were collected and subjected to SDS-PAGE. After transfer to polyvinylidene fluoride membrane, proteins were blocked in TBST containing 5% nonfat dry milk for 1 h at room temperature. Primary and secondary antibody incubations were performed in TBST containing 5% nonfat dried milk at 4 °C for overnight and at room temperature for 1 h, respectively. Three 5-min washes were performed in TBST after the incubations. The membranes were scanned and quantitated with the Odyssey Infrared Imaging System (Li-Cor Biosciences). Statistical analyses were evaluated by one-way ANOVA.

### Quantitative RT-PCR

RNA was isolated from sciatic nerves using the Trizol/chloroform RNA extraction protocol. To prepare cDNA, 250 ng or 1 μg of total RNA of mouse, respectively, was used from each sample. qRT-PCR and data analysis were performed as described previously. qPCR was performed with two replicates per sample, and statistical analyses were evaluated by one-way ANOVA.

### RNA sequencing

About 500 to 1000 ng total RNA was used to generate RNA-Seq libraries using the Illumina TruSeq Stranded total RNA sample preparation kit according to the manufacturer's instructions. Illumina sequencing data were mapped to the GRCm38/mm10 genome. Data were analyzed using DESeq2 (60) to determine differentially regulated genes between uninjured and injured nerves in wildtype and double cKO mice (*p* value < 0.05).

### ChIP sequencing

Sham and injured sciatic nerves of two adult male Sprague–Dawley rats were used in ChIP-seq analysis after micrococcal nuclease digestion of peripheral nerve chromatin as described (13) using an antibody for H2AK119ub1. Library preparation and sequencing was performed by the UW Biotechnology Center as described (11). Basecalling was performed using the standard Illumina Pipeline. Reads were mapped to the *Rattus norvegicus* genome rn5 using Bowtie (RRID:SCR_005476) to produce SAM files for further analysis. Hypergeometric optimization of motif enrichment (HOMER, RRID:SCR_010881) (61) was used to determine enriched binding regions for H2AK119ub1 relative to sequencing of an input chromatin sample.

## Data availability

The raw data files for ChIP-seq are deposited in National Center for Biotechnology Information as part of BioProject PRJNA260442. Data for H3K4me3, H3K27me3, and H2AK119ub1 ChIP analysis were from NCBI Gene Expression Omnibus: GSE84272, GSE84265, and GSE159265. RNA-Seq data are deposited in NCBI GEO under accession number GSE178872.

## Supporting information

This article contains supporting information.

## Conflict of interest

The authors declare that they have no conflicts of interest with the contents of this article.
