# Supplementary material for: H3K27 demethylases are dispensable for activation of Polycomb-regulated injury response genes in peripheral nerve
Source: J Biol Chem. 2021 Jun 4;297(1):100852. doi: 10.1016/j.jbc.2021.100852 (PMC8258988; doi:10.1016/j.jbc.2021.100852)
Supplement: Supplemental Figures S1–S6 [file mmc1.pdf]

Supporting Information

Supplementary Figure 1. JMJD3 Antibody Specificity Testing. Western blot analysis of lysates from S16 cell culture that is knock down with siJMJD3 for 2 d or 3 d after was performed using the indicated antibodies

Supplementary Figure 2. No hyperproliferation nor prolonged proliferation is detected in DDKO after 2 weeks nerve injury. Ki67, a cell proliferation marker, was used to probe the sections of control and DDKO sciatic nerves at 14d after injury.

Supplementary Figure 3. Expression analysis of control and *Jmjd3* cKO mice at 1 3, and 7 DPI. A and B. *Jmjd3* cKO injury dataset indicate no significant differences besides Fgf5 at 3 dpi, which is the only gene found in that of DKO. At 3 dpi, they have recovered in 4 dpi DKO which is similarly seen here in 3 dpi *Jmjd3* cKO data. **C.** No significant difference is seen for many nerve injury genes in *Jmjd3* cKO at 7 dpi with the exception of *Fgf5*. Data: 1 DPI *Jmjd3 cKO* control n=5 and mutant n=5. 3 dpi *Jmjd3 cKO* n=6 and mutant n=6. 7dpi *Jmjd3* cKO control n=6 and mutant n=6. mean ± STDEV; $^{**}p < 0.005$, $^{*}p < 0.05$ (one-way ANOVA).

Supplementary Figure 4. RNA-seq analysis of control and DKO mice at 1 and 7dpi after nerve injury. A. Volcano plots generated from DEseq2 for intact and injury dataset. **B**. Tables summarize the changes of nerve injury and other genes between intact, 1, and 7 days after injury.

Supplementary Figure 5. *Jmjd3* mutant mice resulted in a reduced loss of H3K27me3 levels after nerve injury compared to control. ChIP analysis was performed using lysates from distal stumps and contralateral sham nerve of control and *Jmjd3* cKO sciatic nerves 1 day post cut. 6 pooled nerves for control and *Jmjd3* cKO were used for each of 3 replicate assays. Error bars=S.E.M.

Supplementary Figure 6. Plotting of the average H2AK119ub1 distribution between sham and 1dpi in wildtype based on the list of 343 Polycomb-regulated injury genes. The plot showed that the average distribution is lower in 1dpi compared to that of control.

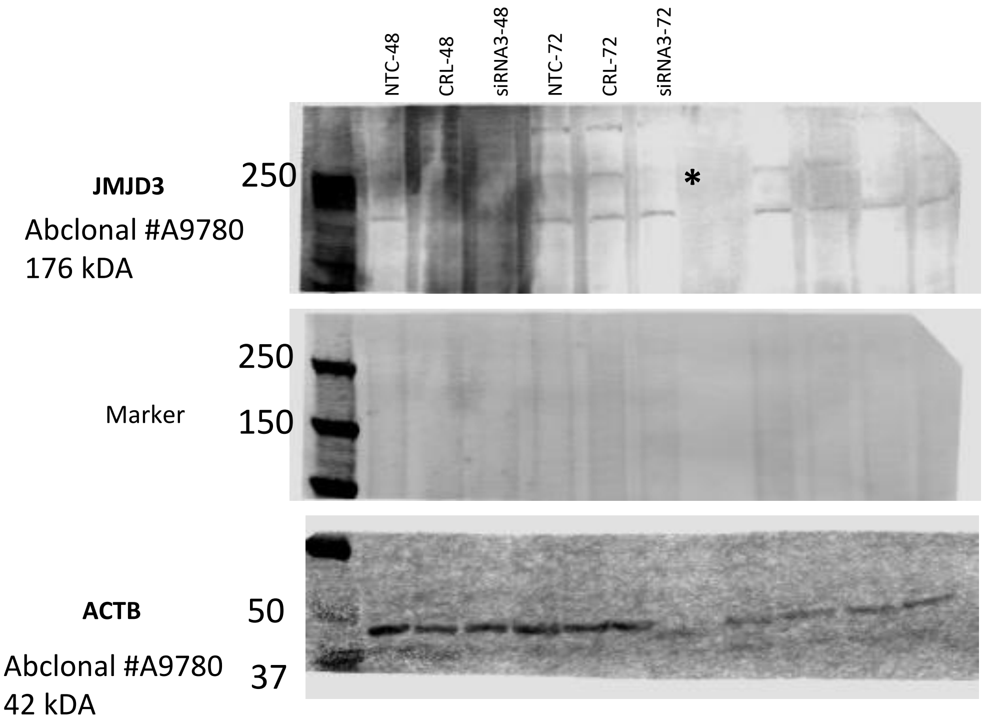

Supplementary Figure 1. JMJD3 Antibody Specificity Testing. Western blot analysis of lysates from S16 cell culture that is knock down with siJMJD3 for 2 d or 3 d after was performed using the indicated antibodies. Top two panels were from same gel (green and red channels of IR imaging), and samples for ACTB were run on a separate gel/blot.

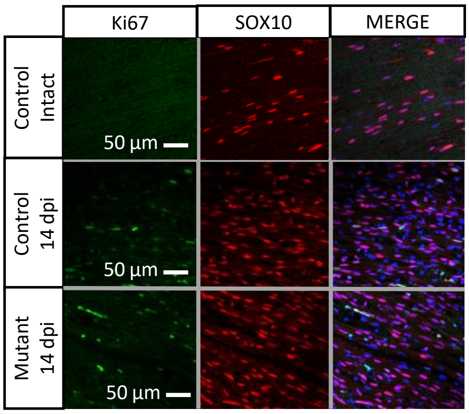

Supplementary Figure 2. No hyperproliferation nor prolonged proliferation is detected in DKO after 2 weeks nerve injury. Ki67, a cell proliferation marker, was used to probe the sections of control and DKO sciatic nerves at 14d after injury.

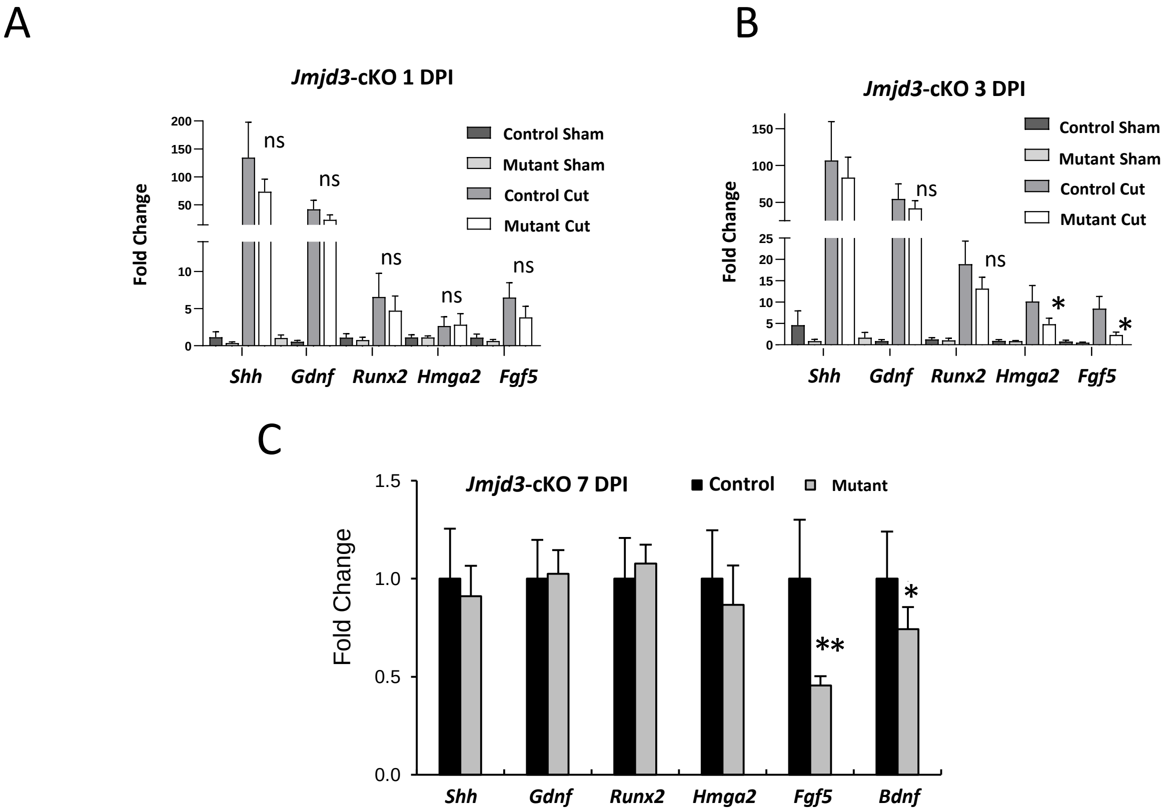

Supplementary Figure 3. Expression analysis of control and *Jmjd3* cKO mice at 1 3, and 7 dpi. A and B. *Jmjd3* cKO injury dataset indicate no significant differences besides *Fgf5* at 3 dpi, which is the only gene found in that of DKO. At 3 dpi, they have recovered in 4 dpi DKO which is similarly seen here in 3 dpi *Jmjd3* cKO data. **C.** No significant difference is seen for many nerve injury genes in *Jmjd3* cKO at 7 DPI with the exception of *Fgf5*. Data: 1 dpi *Jmjd3 cKO* control n=5 and mutant n=5. 3 dpi *Jmjd3 cKO* n=6 and mutant n=6. 7dpi *Jmjd3* cKO control n=6 and mutant n=6. mean ± STDEV; $^{**}p < 0.005$, $^{*}p < 0.05$ (one-way ANOVA).

A

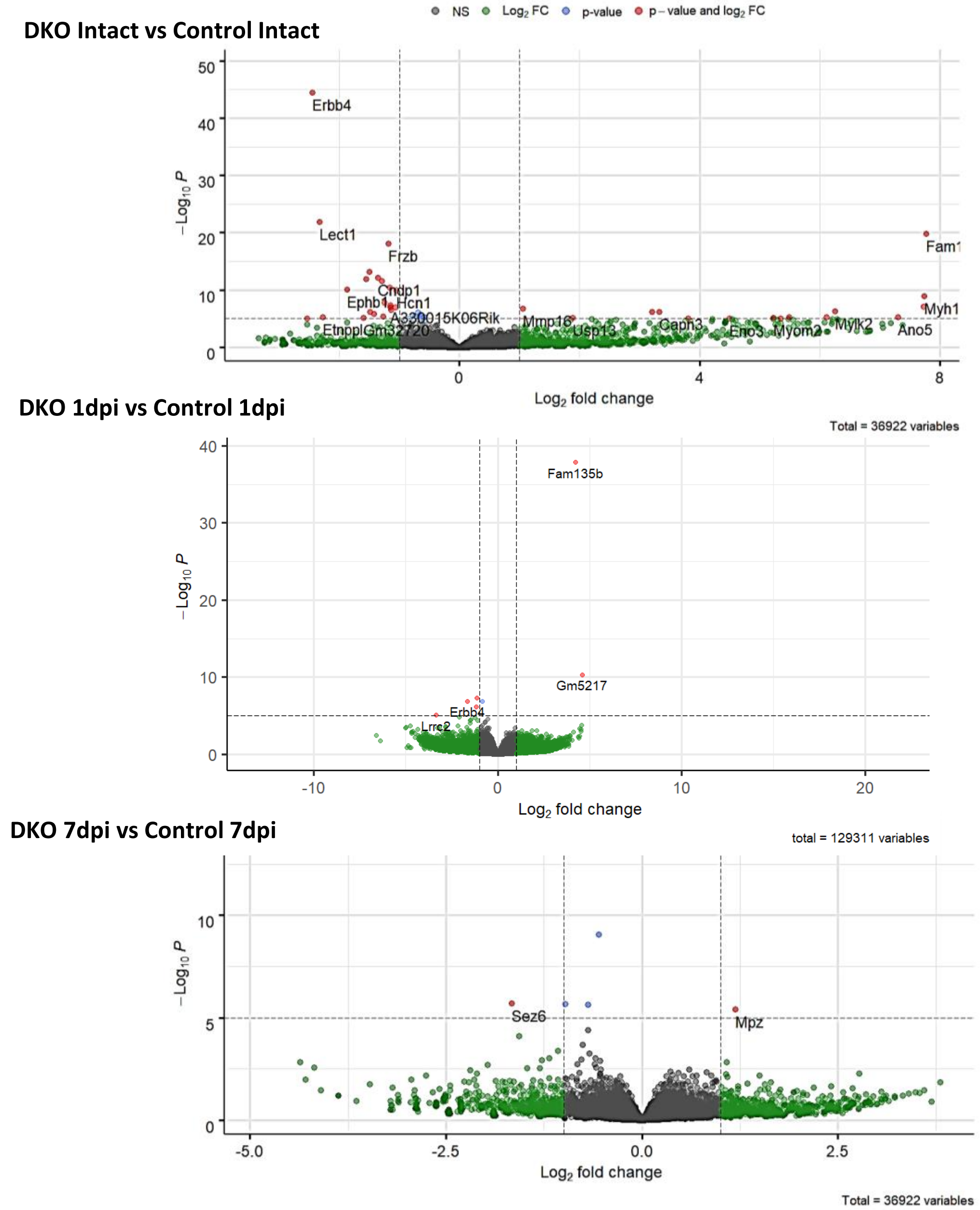

B

Myelin genes

	DE in intact?	DE in 1dpi?	DE in 7dpi?
Pmp22	No	No	No
Mpz	No	No	Yes
Mag	No	No	No
Mbp	No	No	No
Plp1	No	No	No
Egr2	No	No	No
Ndrg1	No	No	No
Cntf	No	No	No
Sox10	No	No	No
Dhh	No	No	No
Il16	No	No	No
Aatk	No	No	No

Polycomb genes

	DE in intact?	DE in 1dpi?	DE in intact?
Gdnf	No	No	No
Olig1	No	No	Yes
Shh	No	No	No
Bdnf	No	No	No
Runx2	No	No	No
Hmga 2	No	No	No
Fgf5	No	Yes	No
Tmpr5 ss	No	No	No
Ccl2	No	No	No
Gfap	No	No	No
Ucn2	No	No	No

Proliferation genes

	DE in intact?	DE in 1dpi?	DE in 7dpi?
Cdkn2a	No	No	No
PCNA	No	No	No
MCM2	No	No	No
Ki67	No	No	No
Pax3	No	No	No
ErbB2	No	No	No
Tgf-beta	No	No	No
Notch	No	No	No
Gpr126	No	No	No
Lamin	No	No	No

Immune genes

	DE in intact?	DE in 1dpi?	DE in 7dpi?
Cd68	No	No	No
Cd11b	No	No	No
Iba1	No	No	No
Arg1	No	No	No
TNF-alpha	No	No	No
Il4	No	No	No
Il1-beta	No	No	No
IL10	No	No	No

Supplementary Figure 4. RNA-seq analysis of control, DKO mice at 1 and 7dpi after nerve injury. A. Volcano plots generated from DEseq2 for intact and injury dataset indicate no overall changes at both 1 and 7 dpi after filtering with cell sort data. **B**. Tables summarize the changes of nerve repair and other genes between intact, 1, and 7 days after injury.

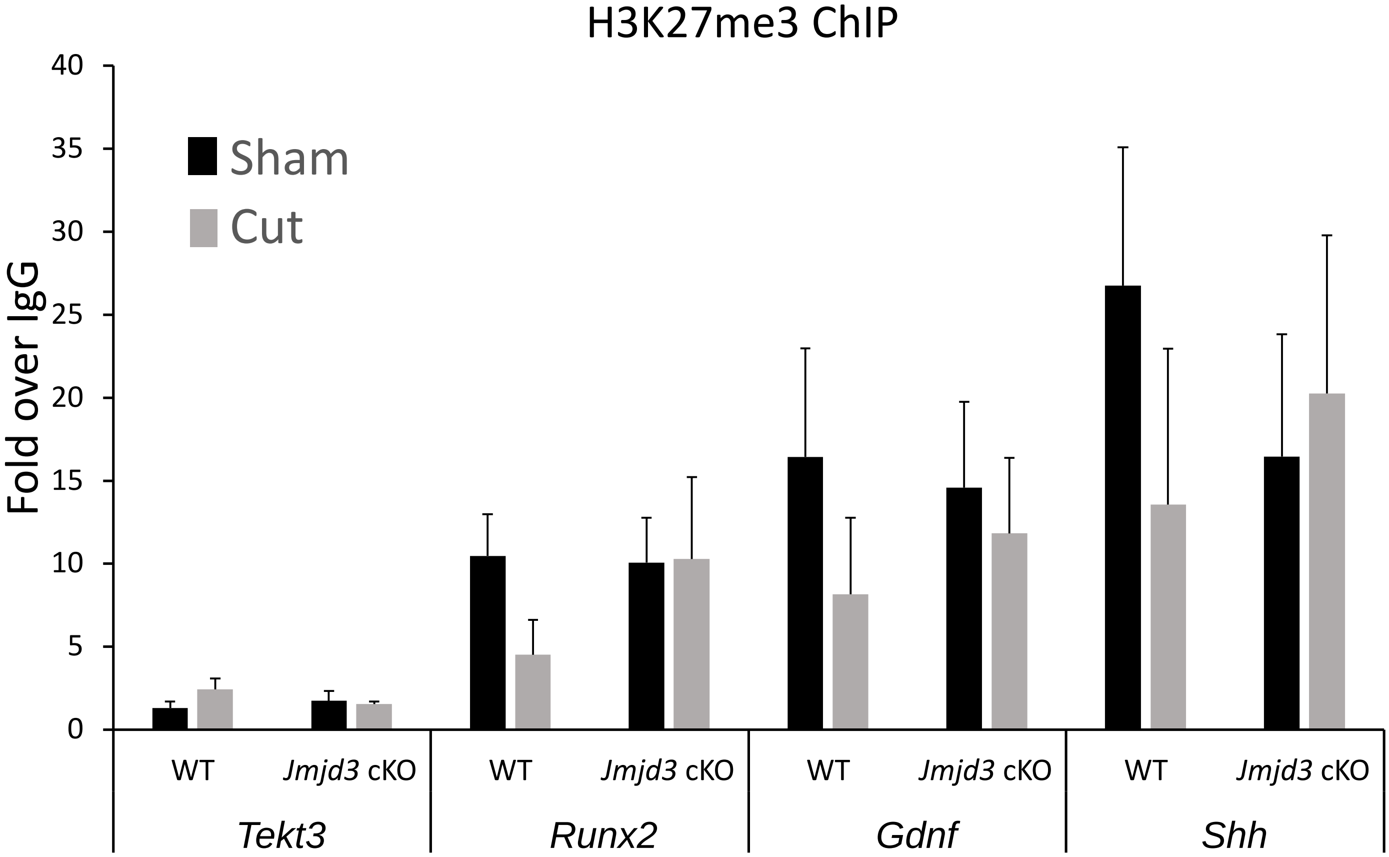

Supplementary Figure 5. *Jmjd3* mutant mice resulted in a reduced loss of H3K27me3 levels after nerve injury compared to control. ChIP analysis was performed using lysates from distal stumps of control and *Jmjd3* cKO sciatic nerves 1 day post cut. 6 pooled nerves for control and *Jmjd3* cKO were used for each of 3 replicate assays. Error bars=S.E.M.

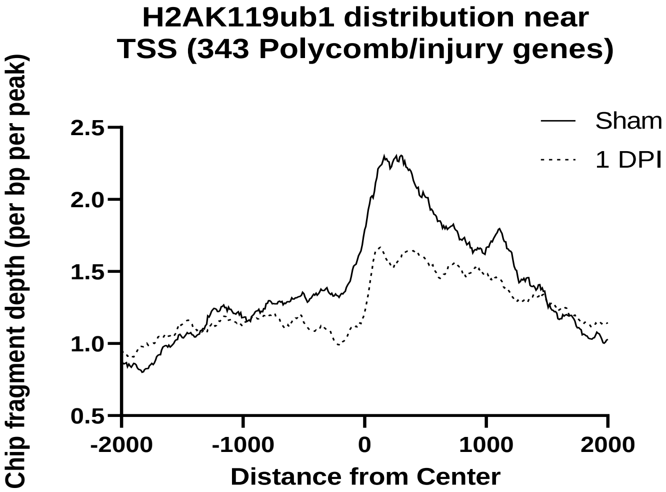

Supplementary Figure 6. Plotting of the average H2AK119ub1 distribution between sham and 1dpi in wildtype based on the list of 343 Polycomb-regulated injury genes. The plot showed that the average distribution is lower in 1dpi compared to that of sham.
